# Eating Vegetables First Regardless of Eating Speed Has a Significant Reducing Effect on Postprandial Blood Glucose and Insulin in Young Healthy Women: Randomized Controlled Cross-Over Study

**DOI:** 10.3390/nu15051174

**Published:** 2023-02-26

**Authors:** Saeko Imai, Shizuo Kajiyama, Kaoru Kitta, Takashi Miyawaki, Shinya Matsumoto, Neiko Ozasa, Shintaro Kajiyama, Yoshitaka Hashimoto, Michiaki Fukui

**Affiliations:** 1 Department of Food and Nutrition, Faculty of Home Economics, Kyoto Women’s University, 35, Kitahiyoshi-cho, Imakumano, Higashiyama-ku, Kyoto 605-8501, Japan; 2 Kajiyama Clinic, Kyoto Gojyo Clinic Building 20-1, Higasionnmaeda-cho, Nishinanajyo, Shimogyo-ku, Kyoto 600-8898, Japan; 3 Graduate School of Medical Science, Kyoto Prefectural University of Medicine, 465 Kajii-cho, Kawaramachi-Hirokoji, Kamigyo-ku, Kyoto 602-8566, Japan; 4 Graduate School of Medicine, Kyoto University, 54, Kawahara-cho, Syogoin, Sakyo-ku, Kyoto 606-8507, Japan; 5 Japanese Red Cross Kyoto Daini Hospital, 355-5, Kamanza, Marutamachi, Kamigyo-ku, Kyoto 602-8026, Japan

**Keywords:** diet, eating speed, food order, postprandial blood glucose, insulin, type 2 diabetes, vegetable, carbohydrate

## Abstract

People with fast eating habits have been reported to have an increased risk of diabetes and obesity. To explore whether the speed of eating a test meal (tomato, broccoli, fried fish, and boiled white rice) influences postprandial blood glucose, insulin, triglyceride, and free fatty acid levels, 18 young, healthy women consumed a 671 kcal breakfast at fast speed (10 min) and slow speed (20 min) with vegetables first and slow speed (20 min) with carbohydrate first on three separate days. This study was conducted using a within-participants cross-over design in which all participants consumed identical meals of three different eating speeds and food orders. Significant ameliorations of both fast and slow eating with vegetables first regimen on postprandial blood glucose and insulin levels at 30 and 60 min were observed compared with those of slow eating with carbohydrates first. In addition, the standard deviation, large amplitude of excursion, and incremental area under the curve for blood glucose and insulin in both fast and slow eating with vegetables first were all significantly lower than those of slow eating with carbohydrate first. Interestingly, there was no significant difference between fast and slow eating on postprandial blood glucose and insulin levels as long as vegetables were consumed first, although postprandial blood glucose at 30 min was significantly lower in slow eating with vegetables first than that of fast eating with the same food order. These results suggest that food order with vegetables first and carbohydrate last ameliorates postprandial blood glucose and insulin concentrations even if the meal was consumed at fast speed.

## 1. Introduction

The number of people with diabetes is currently estimated to be as many as 537 million, and it is predicted to increase up to 783 million by 2045, with more than 90% of them estimated to be type 2 diabetes (T2DM) [1]. Diabetes is known to be responsible for the development of various complications. For example, diabetes is the leading cause of renal failure, new onset blindness, and lower-extremity amputation in the United States [2]. The goal of treatment for T2DM is centered on preventing or delaying complications and maintaining quality of life for the patient, and those goals are achieved by the appropriate management of hyperglycemia according to the American Diabetes Association (ADA) and European Association for the Study of Diabetes (EASD) [3].

Postprandial blood glucose elevation and mean amplitude of glycemic excursions (MAGE) are associated with the pathogenesis of micro- and macrovascular complications in individuals with diabetes and the incidence of T2DM [4,5,6]. Therefore, reducing postprandial blood glucose and glycemic excursions are the key strategies of pharmacological and medical nutrition therapy for preventing diabetic complications and reducing the risk of T2DM. Above all, diet is the leading contributor in the management and prevention of T2DM [7]. Many dietary approaches are proven effective and available, such as the Mediterranean diet [8], low-calorie diet [9], low-carbohydrate diet [10], low-glycemic-index diet [11], vegetarian diet [12], and intermittent fasting diet [13]. The effectiveness of these diets has been demonstrated but some difficulties remain. Despite that medical nutrition therapy should continue as a lifelong remedy, long-term interventional studies are limited. For example, the Dietary Intervention Randomized Controlled Trial (DIRECT) study was a well-known trial that compared the long-term effect of three dietary methods, the Mediterranean diet, low-fat diet, and low-carbohydrate diet, on obese individuals for 6 years including a 4-year follow-up. The results demonstrated that the Mediterranean and low-carbohydrate diet were more effective than the low-fat diet on weight loss and lipid profiles after 2 years, although, after 6 years, 11% of the participants had changed to another diet, and surprisingly, 22% of the participants quit dieting [14]. This research reveals that it is difficult to continue any dietary methods for a long term of more than 2 years.

Recent studies including our research indicate that the food order of preloading vegetables, protein, or fat with slow eating can ameliorate postprandial blood glucose excursions and decrease insulin secretion in both individuals with and without T2DM [15,16,17,18]. Nowadays, the food order, as an innovative medical nutrition therapy, is widely recognized as being effective for individuals with and without T2DM in Japan, and significant effects on acute and chronic glycemic control have been demonstrated in individuals with T2DM [19,20].

On the other hand, epidemiological and cohort studies demonstrated that fast eating speed resulted in weight gain [21,22,23] and increased incidence of T2DM and metabolic syndrome [24,25,26]. These studies demonstrated that the modification of eating speed could be an efficient and cost-effective method for promoting weight management in obese and healthy individuals to prevent the incidence of T2DM. However, people who were instructed to eat slowly had difficulty in accomplishing the task [27]. Therefore, instead of a slowing eating pace, strategies of different aspects are needed to improve postprandial blood glucose and insulin responses for preventing obesity and T2DM.

We previously reported that eating fast (10 min) with a mixed eating of vegetables, protein, and carbohydrate demonstrated significant higher glycemic excursions compared with eating slow (20 min) with vegetables first in young healthy women [28]. However, the effect of eating fast with vegetables first on postprandial blood glucose and insulin concentrations is still uncertain. The purpose of this study was to examine the acute effect of different eating speeds with different food orders on postprandial blood glucose, insulin, triglyceride (TG), and free fatty acid (FFA) concentrations in young healthy women.

## 2. Methods

### 2.1. Study Design

Participants were enrolled from volunteers from the Kyoto Women’s University, Kyoto, Japan. All volunteers were informed of the purpose, protocol, and risks of the research before the study began. Twenty-one participants were registered in the study. Written informed consents were obtained from all participants. The study was conducted between April 2022 and July 2022. None of the participants were pregnant or smokers. The participants were also free from eating disorders, metabolic diseases, other diets, and any medications and supplements identified to affect their blood glucose, insulin, and lipid levels.

**Figure 1:**
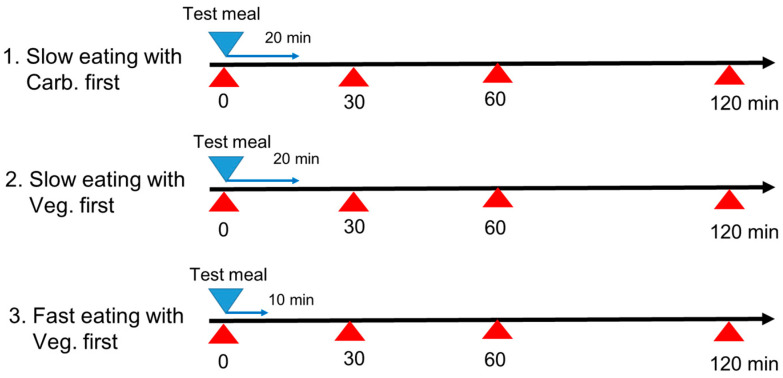
Study protocol. All participants consumed identical test meals in 3 different eating patterns: eating slow (20 min) with carbohydrate first, eating slow (20 min) with vegetables first, and eating fast (10 min) with vegetables first. Each meal was consumed at 9:00 under the following experimental conditions that were assigned in the unblind randomized cross-over trial: 1. Carbohydrate first with slow eating speed: carbohydrate (boiled white rice) first for 6 min, and then protein (fried fish) for 7 min, and then vegetables (tomato and broccoli with sesame oil) for 7 min, for a total eating time of 20 min. 2. Vegetables first with slow eating speed: vegetables (tomato and broccoli with sesame oil) first for 7 min, and then protein (fried fish) for 7 min, and then carbohydrate (boiled white rice) for 6 min, for a total eating time of 20 min. 3. Vegetables first with fast eating speed: vegetable (tomato and broccoli with sesame oil) first for 4 min, and then protein (fried fish) for 3 min, and then carbohydrate (boiled white rice) for 3 min, for a total eating time of 10 min. Blood samples were collected at 0, 30, 60, and 120 min after consuming the test meals. Postprandial blood glucose, insulin, triglyceride (TG) and free fatty acid (FFA) concentrations were examined. Veg.; vegetables, Carb.; carbohydrate.

This study was conducted using an unblind randomized within-participants cross-over design in which all participants consumed identical meals on three separate days. The study protocol relating to human subjects was approved by the Ethics Committee of Kyoto Women’s University (2021-21) according to the guidelines of the Declaration of Helsinki. The study protocol was registered in the UMIN Clinical Trials Registry (UMIN000050266). Each participant consumed identical test meals on three separate days, and each study day was 1 week apart with three different eating patterns of fast speed and slow speed with vegetables first and slow speed with carbohydrate first, as shown in Figure 1.

1. Carbohydrate first with slow eating speed: carbohydrate (boiled white rice) first for 6 min, and then protein (fried fish) for 7 min, and then vegetables (tomato and broccoli with sesame oil) for 7 min, for a total eating time of 20 min.
2. Vegetables first with slow eating speed: vegetables (tomato and broccoli with sesame oil) first for 7 min, and then protein (fried fish) for 7 min, and then carbohydrate (boiled white rice) for 6 min, for a total eating time of 20 min.
3. Vegetables first with fast eating speed: vegetables (tomato and broccoli with sesame oil) first for 4 min, and then protein (fried fish) for 3 min, and then carbohydrate (boiled white rice) for 3 min, for a total eating time of 10 min.

**Figure 2:**
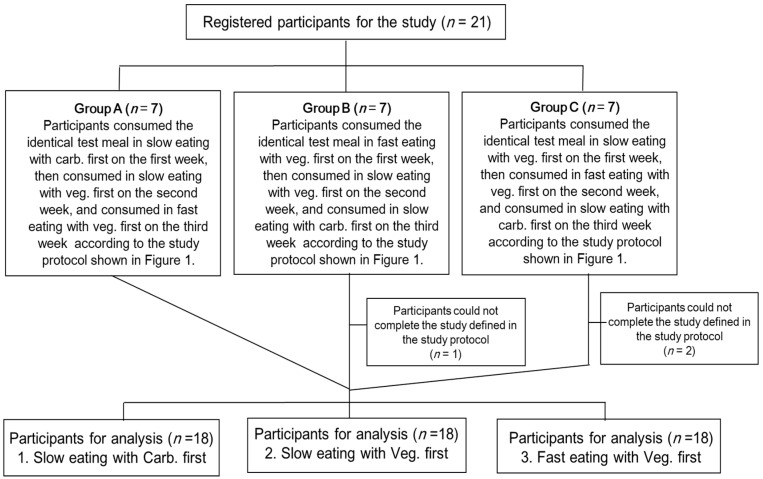
Study flow. Registered participants were divided into 3 groups with 7 participants each. All participants consumed the identical test meals for three days according to the study protocol shown in Figure 1. Group A: Participants consumed the identical test meal in slow eating with carb. First on the first week, then consumed in slow eating with veg. first on the second week, and consumed in fast eating with veg. first on the third week. Group B: Participants consumed the identical test meal in fast eating with veg. first on the first week, then consumed in slow eating with veg. first on the second week, and consumed in slow eating with carb. First on the third week. Group C: Participants consumed the identical test meal in slow eating with veg. first on the first week, then consumed in fast eating with veg. first on the second week, and consumed in slow eating with carb. First on the third week. Veg.; vegetables, carb.; carbohydrate.

The study flow is shown in Figure 2. Twenty-one participants were divided into 3 groups with 7 participants each. All participants consumed identical test meals for three days according to the study protocol. The allocation sequence of Group A, B, and C was assigned by the research members in order of the research ID number of each participant. Group A: Participants consumed the identical test meal in slow eating with carbohydrate first on the first week, then consumed in slow eating with vegetables first on the second week, and consumed in fast eating with vegetables first on the third week. Group B: Participants consumed the identical test meal in fast eating with vegetables first on the first week, then consumed in slow eating with vegetables first on the second week, and consumed in slow eating with carbohydrate first on the third week. Group C: Participants consumed the identical test meal in slow eating with vegetables first on the first week, then consumed in fast eating with vegetables first on the second week, and consumed in slow eating with carbohydrate first on the third week.

On the study day, the participants arrived at 8:30 at the Kyoto Women’s University after a 12 h overnight fast, and each meal was consumed at 9:00 under the experimental conditions that were randomly assigned to the participants (Figure 2). Blood samples were collected by the nurse at Kyoto Women’s University at 0, 30, 60, and 120 min after test meal consumption (Figure 1). Postprandial blood glucose, insulin, TG, and FFA concentrations were examined. The area under the curve (IAUC) measurements for glucose and insulin concentrations were calculated by the trapezoidal method above the baseline concentration at 9:00 and 120 min after consuming the test meals. The concentrations of postprandial blood glucose, insulin, TG, and FFA were compared within the participants among the three study days.

### 2.2. Meals for the Study

The macronutrient amounts of the test meal (63% from carbohydrate, 15% from protein, 22% from fat) are shown in Table 1. The frozen bento of fried fish (Tokatsu Foods, Yokohama, Japan), vegetables (150 g of tomato and 70 g of broccoli), and 200 g of boiled white rice were purchased and served to the participants as breakfast by the research members. The frozen bento boxes were kept in the freezer until consumption and heated with a microwave by researchers on the test days before consumption. The participants consumed test meals with 200 g of water. The researchers measured and recorded eating speed and food order and assessed for compliance of the study protocol. The participants who did not follow the protocol were excluded.

### 2.3. Primary and Secondary Measurements and Statistical Analysis

On the first day of the study, anthropometric measurements of height and body weight, and the hemoglobin A1c (HbA1c) of the participants were measured at Kyoto Women’s University in the morning after an overnight fast. All blood samples were examined by Nihon Rinsho, Inc, Kyoto, Japan. Plasma glucose concentration was measured by HK-G6PDH methods (KANTO CHEMICAL CO., INC. Tokyo, Japan). HbA1c levels were determined by NGSP method (Kyowa Medix CO., INC, Tokyo, Japan). Serum insulin levels were determined by CLEIA method (FUJIREBIO. CO., INC. Tokyo, Japan). Serum TG concentrations were determined by GK-GPO method (SEKISUI MEDICAL CO., LTD. Tokyo, Japan). Serum FFA concentrations were determined by ACS-ACOD method (FUJIFILM Wako Pure Chemical Corporation, Osaka, Japan).

In the present study, a total of fourteen participants was calculated as the sample size to provide 95% power to detect 5% difference in postprandial blood glucose levels (G*Power 3.1, Heinrich-Heine-Universität Düsseldorf, Germany), matched to our study of consuming test meals of different food order in young, healthy women [29]. The primary outcome was the postprandial blood glucose concentration and the secondary outcome was the postprandial blood insulin concentration. Since we could not statistically confirm homogeneity of variance and normal distribution for all blood parameters by Shapiro–Wilk and Levene’s tests, we used a paired comparison by the Wilcoxon matched-pairs signed-rank test followed by post hoc Bonferroni’s inequality when Friedman’s test revealed significant effects for parameters (*p* < 0.05). The results are expressed as the mean ± standard error of the mean (SEM) unless otherwise stated. All analyses were performed with SPSS Statistics ver. 24 software (IBM Corp., Armonk, NY, USA).

## 3. Results

**Figure 3:**
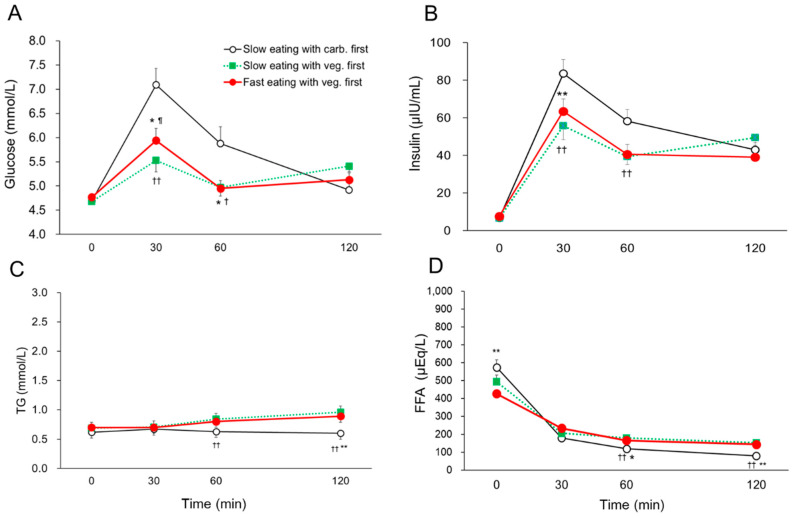
The postprandial blood glucose (**A**), insulin (**B**), TG (**C**), and FFA (**D**) concentrations at 0, 30, 60, and 120 min in the three different study days on which identical meals were consumed with different eating speed and food order in young, healthy women (*n* = 18). Data are mean ± SEM. TG; triglyceride, FFA; free fatty acid. veg.; vegetables, carb.; carbohydrate. Slow eating with carb. first vs. Fast eating with veg. first; *p* < 0.05 *, *p* < 0.01 **. Slow eating with carb. first vs. Slow eating with veg. first; *p* < 0.05 ^†^, *p* < 0.01 ^††^. Slow eating with veg. first vs. Fast eating with veg. first; *p* < 0.05 ^¶^.

Among the 21 participants, 3 participants could not complete the study defined by the study protocol, so the results were analyzed based on 18 young, healthy women [age 21.3 ± 0.4 years, BMI 19.6 ± 1.6 kg/m^2^, FPG 4.7 ± 0.3 mmol/L, HbA1c 33 ± 2 mmol/mol (5.2 ± 0.3%), mean ± standard deviation (SD), Figure 2]. None of the participants had a family history of T2DM. The postprandial blood glucose, insulin, TG, and FFA profiles for three different days with different food orders and eating speeds are shown in Figure 3. The significant amelioration of both fast and slow eating with vegetables first on postprandial blood glucose at 30 min (slow eating with carb. first vs. fast eating with veg. first; 7.09 ± 0.34 vs. 5.94 ± 0.24 mmol/L, *p* < 0.05. vs. slow eating with veg. first; 5.53 ± 0.25 mmol/L, *p* < 0.01, mean ± SEM) and 60 min (slow eating with carb. first vs. fast eating with veg. first; 5.88 ± 0.34 vs. 4.95 ± 0.18 mmol/L, vs. slow eating with veg. first; 4.97 ± 0.16 mmol/L, both *p* < 0.05) was demonstrated compared with those in slow eating with carbohydrate first (Figure 3A). Additionally, postprandial blood insulin concentrations at 30 min (slow eating with carb. first vs. fast eating with veg. first; 83.6 ± 7.5 vs. 63.5 ± 7.5 μIU/mL, vs. slow eating with veg. first; 55.8 ± 6.5μIU/mL, both *p* < 0.01) and 60 min (slow eating with carb. first vs. slow eating with veg. first; 58.2 ± 6.4 vs. 39.4 ± 5.2 μIU/mL, *p* < 0.01) were significantly lower in slow and/or fast eating with vegetables first than those of slow eating with carbohydrate first (Figure 3B). In contrast, postprandial blood FFA concentrations decreased after consuming the test meal in the three days. Postprandial blood TG and FFA concentrations at 60 min and 120 min in slow eating with carbohydrate first showed lower values than those of slow and/or fast eating with vegetables first; yet, pre- and postprandial blood TG and FFA concentrations were transit within the normal range in all three study days (Figure 3C,D).

Blood glucose and insulin parameters of different speeds with different food orders were shown in Table 2. The significant reductions of both fast and slow eating with vegetable first on SD, large amplitue of glucemic excursion (LAGE), and IAUC 120 min for blood glucose were observed compared with those in slow eating with carbohydrate first. Additionally, SD, MAX, large amplitude of insulin excursion, and IAUC 120 min for insulin in both fast and slow eating with vegetable first were significantly lower than those of slow eating with carbohydrate first. However, there was no significant difference between fast and slow eating with vegetable first on postprandial blood glucose and insulin, except postprandial blood glucose at 30 min in slow eating with vegetable first was significantly lower than that of fast eating with vegetable first (Table 2 and Figure 3A).

## 4. Discussion

This is the first interventional study to demonstrate that fast eating with vegetables first ameliorates postprandial blood glucose elevation and insulin secretion in young, healthy women. Our present study indicates that as long as vegetables are consumed first, eating speed, whether slow (20 min) or fast (10 min), does not affect postprandial blood glucose and insulin levels, except slow eating with vegetables first showed lower postprandial blood glucose concentration at 30 min than that of fast eating with the same food order. It is important to “eat vegetables first and carbohydrate last” to ameliorate postprandial blood glucose and insulin even in fast eating, as supported by our previous study showing that eating fast with a mixed eating with vegetable, protein, and carbohydrate elevated postprandial blood glucose compared with slow eating with vegetables first [28].

As the test meals were provided in the morning after 12 h fasting, postprandial blood FFA concentrations decreased after consumption of the meal in all three days, while postprandial blood glucose and insulin secretion increased after consuming the meal. This seems reasonable because the postprandial blood FFA concentrations were inversely correlated to the blood glucose concentration. Thus, the postprandial blood FFA concentrations in slow eating with carbohydrate first were lower than those of slow and fast eating with vegetables first. The reason of higher postprandial blood TG concentrations shown in slow and fast eating with vegetables first compared with those in slow eating with carbohydrate first might be explained by the sesame oil consumed first with vegetables. Although postprandial parameters of blood TG and FFA in fast and slow eating with vegetables first showed statistically higher than the values in slow eating with carbohydrate first, all of the values were within the normal range of a healthy population without dyslipidemia, suggesting these variations were normal, rather than any pathological phenomena.

Shukla et al. reported that a food order with vegetables for 10 min, followed by a 10 min interval, and then eating protein and carbohydrate for 10 min was effective to suppress postprandial blood glucose elevation and insulin secretion [16]. However, in the present study, we demonstrated the effect of eating fast for 10 min with a food order of vegetables, protein, and carbohydrate without any interval time. Kuwata et al. reported that eating protein first ameliorated postprandial blood glucose, although it did not suppress insulin secretion [30]. This fact is particularly important for Japanese individuals with and without T2DM because the secretion of insulin in East Asian people including Japanese is often delayed, and the ability for insulin secretion is weak, about half that of Caucasian’s [31]. Thus, for East Asians, it is essential to suppress excessive insulin secretion to maintain β cell function and potentially lower the risk of obesity, T2DM, cancer [32], and Alzheimer’s disease [33].

The food order and eating speed in the present study, i.e., “eating vegetables first, then the main dish (protein), and then carbohydrate last for 10 min”, is easier to maintain in real life than other methods of medical nutrition therapy for T2DM. For instance, we have reported previously that the dietitian-led medical nutrition therapy of food order with vegetables first has been reported to be effective in the long term, up to 5 years, on glycemic control and the prevention of diabetic complications in individuals with T2DM [19,34].

One of the possible reasons for the amelioration of postprandial blood glucose and insulin concentration observed in fast eating with vegetables first may be explained by the preloading of dietary fiber contained in vegetables (7.1 g of dietary fiber in the test meal). The dietary fiber in the test meal was digested slowly to ameliorate postprandial blood glucose elevation and reduce insulin secretion [35,36]. Another possibility in the present study is that the secretion of incretin hormones, such as glucagon-like peptide-1 (GLP-1) and glucose-dependent insulinotropic polypeptide (GIP), might have been induced by the sesame oil consumed with the vegetables. Then, consuming the main dish which contains protein and fat may have induced incretin hormones. Incretin hormones induce insulin secretion for subsequent metabolic responses and delayed gastric emptying. The delay of gastric emptying by incretin hormones was reported to ameliorate postprandial blood glucose elevation and minimize the enhancement of glucose-dependent insulin secretion [17,18,20,30].

Various cohort and epidemiological studies revealed that a faster eating speed showed a positive correlation with body weight, blood glucose concentration, insulin resistance, and the risk of metabolic syndrome and T2DM [21,22,23,24,25,26]. However, the slowing of eating pace was not an easy task to achieve [27]. Therefore, instead of changing eating speed, strategies from different aspects are needed to improve postprandial blood glucose and insulin responses. In addition, these epidemiological studies were not interventional studies; they were analyzed through surveys reported by the subjects themselves, which might cause bias in assessing the effect of eating speed on metabolic parameters.

On the other hand, some research has reported that eating speed does not affect postprandial blood glucose, insulin, and incretin hormone levels in individuals with and without T2DM [37,38]. Moreover, not only eating speed but also specific dietary patterns are associated with an increased risk of T2DM, obesity, and metabolic syndrome. The dietary patterns in individuals who eat fast tended to be Western-style eating patterns high in red meat, processed meat, snacks, sweetened beverages, fried food, and fast-food, and low in vegetables [39]. In addition, eating quickly often resulted in higher energy intake by increasing appetite and hunger [21,22,23,24,25,26,40]. Thus, eating quickly may increase the risk of obesity and insulin resistance, and subsequently worsen the metabolic responses.

Various evidence-based studies suggest that a diet rich in vegetables and low GI grains is the substantial dietary pattern predicting low risk in T2DM, obesity, and cardiovascular diseases [11]. We assume that individuals with a dietary pattern of fast eating are likely to avoid fiber-rich food which requires chewing thoroughly and takes a certain time for ingesting [41]. In the present study, boiled white rice with high GI was used because low GI grains such as brown rice and whole-grain bread are unfavorable for Japanese people. Therefore, dietary fiber should be taken from vegetables, seaweeds, mushrooms, soy beans, and soy products in Japan. However, the average vegetable intake was less than the 350 g recommended by the Ministry of Health, Labor and Welfare in Japan [42]. The U.S. report reveals that 70% of individuals in the U.S. do not consume the amount of vegetables recommended by the U.S. Department of Agriculture, and shockingly, 25% of individuals do not consume vegetables at all. [43]. The reasons of low vegetable intake are described to be such as economic problems, absence of knowledge of the benefits of vegetables, low accessibility to fresh products, taste preference, and lack of cooking time and skills. Therefore, we previously demonstrated that the preloading of tomato juice [44] or vegetable juice [45] was effective to reduce postprandial blood glucose concentrations. Tomato and vegetable juice are easy to consume and cost less than consuming fresh vegetables; thus, their preloading is one of the simple and economical methods to ameliorate postprandial blood glucose concentrations.

Furthermore, cost–benefit analysis suggests that dietary education would produce a significant potential cost-saving effect in national healthcare budgets [46]. International treatment costs for diabetes may reach as high as 10% of national health expenditures, and the losses of national income can be equally costly; treatment costs are escalating along with the increasing prevalence of diabetes. Above all, there is concern that drugs alone may not prevent the progression of diabetic complications [47]. Nevertheless, patients with T2DM treated by medical nutrition therapy may be less managed than patients on medication with a list of prescriptions. Therefore, the eating behavior shown in this study should be one of the effective medical nutrition therapies not only to improve metabolic responses but also national healthcare budgets.

Our results indicate that in addition to the amount of energy, carbohydrate, fat, protein, dietary fiber, and other nutrient contents, food order, rather than eating speed, is the most important factor for postprandial blood glucose and insulin responses. It is essential to avoid only eating carbohydrates, such as boiled rice, noodles, and bread, for preventing postprandial blood glucose elevation. Obviously, medical nutrition therapy with food order should require counselling patients to support the individual’s dietary habit, socio-economic situation, and lifestyle, as well as their medical condition.

The current study has limitations to be mentioned. First, the present experiment was designed to examine the acute effects of eating speed with different food order in a single meal, requiring additional investigations to clarify the long-term effect on glycemic control and the improvement of diabetic complications in T2DM management, and the chronic effect of diabetes prevention in individuals without T2DM. Second, the study participants were Japanese young women without T2DM and dyslipidemia; therefore, it is uncertain whether the present results could be applied to individuals with T2DM and dyslipidemia, and of other gender, age groups, or racial groups. Third, incretin hormones may be involved in the mechanisms for amelioration of blood glucose, insulin, and the gastric emptying rate, because enhanced GLP-1 secretion is reported to delay gastric emptying in both individuals with and without T2DM [16,17,19,29]. Although GLP-1, GIP, and gastric emptying rates were not examined in this study, the role of eating fast and food order on the postprandial blood glucose–incretin hormone interaction remains unclear. Therefore, the results of this research should be used carefully. Further investigations are required to determine the overall mechanisms of eating rate and food order on glycemic and hormone responses in individuals with and without T2DM.

## 5. Conclusions

The present study demonstrates that as long as vegetables are consumed first, eating speed, whether slow (20 min) or fast (10 min), does not affect postprandial blood glucose and insulin concentrations when an appropriate amount of vegetable is consumed. Our current results provide more practical evidence for medical nutrition therapy as a real-world approach for the better prevention and management of individuals with and without T2DM.

## Figures and Tables

**Table 1:** Macronutrient contents of the test meal.

	Amount	Energy	Protein	Fat	Carbohydrate	Dietary Fiber	Salt
	(g)	(kcal)	(g)	(g)	(g)	(g)	(g)
Boiled while rice	200	336	5	0.6	74.2	3	0
Tomato	150	29	1.1	0.2	7.1	1.5	0
Broccoli	70	19	2.5	0.3	3	2.6	0
Sesame oil	10	92	0	10	0	0	0
Frozen box of fried fish	237	195	15.9	5.2	21.1	0	2.2
Total	667	671	24.5	16.3	105.4	7.1	2.2

**Table 2:** Blood glucose and insulin parameters of different speeds with different food orders in young, healthy women.

	Slow Eating with Carb. First (*n* = 18)	Slow Eating with Veg. First (*n* = 18)	Fast Eating with Veg. First (*n* = 18)
MBG (mmoll/L)	5.65 ± 0.21	5.15 ± 0.11	5.20 ± 0.12
SD for BG (mmoll/L)	1.06 ± 0.11 *†	0.58 ± 0.06	0.61 ± 0.05
MAX BG (mmoll/L)	7.13 ± 0.33	5.92 ± 0.19	6.10 ± 0.19
LAGE (mmoll/L)	2.71 ± 0.26 *†	1.50 ± 0.14	1.60 ± 0.14
IAUC 120 min for BG (mmoll/L × min)	140 ± 27 *†	68 ± 12	67 ± 12
Mean insulin (μIU/mL)	47.9 ± 3.7	37.9 ± 3.6	37.7 ± 3.7
SD for insulin (μIU/mL)	29.7 ± 2.3 **††	21.0 ± 2.1	21.8 ± 2.3
MAX insulin (μIU/mL)	85.6 ± 7.7 **†	61.8 ± 5.7	65.8 ± 6.9
Large amplitude of insulin excursion (μIU/mL)	78.8 ± 7.3 **††	55.0 ± 5.2	58.2 ± 6.3
IAUC 120 min for insulin (μU/mL × min)	5702 ± 486 *††	4222 ± 458	4113 ± 402

Data are mean ± SEM. MBG; mean blood glucose, BG; blood glucose, SD; standard deviation, LAGE; large amplitude of glucose excursion, IAUC; incremental area under the curve. Slow eating with carb. first vs. Fast eating with veg. first; *p* < 0.05 *, *p* < 0.01 **. Slow eating with carb. first vs. Slow eating with veg. first; *p* < 0.05 †, *p* < 0.01 ††.

## Data Availability

The data are not publicly available due to privacy reasons. Data supporting the reported results are available upon reasonable request and in accordance with the ethical principles.
